# Advances and Current Challenges in Intestinal *in vitro* Model Engineering: A Digest

**DOI:** 10.3389/fbioe.2019.00144

**Published:** 2019-06-18

**Authors:** Joana Costa, Arti Ahluwalia

**Affiliations:** Research Center “E. Piaggio” and Department of Information Engineering, University of Pisa, Pisa, Italy

**Keywords:** intestine, *in vitro* models, bioreactor, 3D scaffolds, tissue engineering

## Abstract

The physiological environment of the intestine is characterized by its variegated composition, numerous functions and unique dynamic conditions, making it challenging to recreate the organ *in vitro*. This review outlines the requirements for engineering physiologically relevant intestinal *in vitro* models, mainly focusing on the importance of the mechano-structural cues that are often neglected in classic cell culture systems. More precisely: the topography, motility and flow present in the intestinal epithelium. After defining quantitative descriptors for these features, we describe the current state of the art, citing relevant approaches used to address one (or more) of the elements in question, pursuing a progressive conceptual construction of an “ideal” biomimetic intestinal model. The review concludes with a critical assessment of the currently available methods to summarize the important features of the intestinal tissue in the light of their different applications.

## Introduction

The study of intestinal phenomena, as well as intestinal disorders, has been empowered by the use of animal models. However, many intestinal processes are difficult to control using *in vivo* models. *In vitro* models are employed to facilitate the study of complex *in vivo* phenomena in a simplified context, allowing well-controlled and repeatable conditions for the evaluation of cell response. They can be used in many different fields thanks to their wide areas of application including toxicology, drug testing, tissue engineering and nutraceutics (Mattei et al., 2014). Moreover, *in vitro* intestinal models can potentially enable improved studies of cellular growth and proliferation, drug absorption, and host-microbial interactions, while reducing the expense and ethical issues associated with the use of animal experiments (Cencič and Langerholc, 2010). In this context, current guidelines and legislation on the use of animals in science adhere to the “3Rs” principles, defined by Russell and Burch (1959); Guidelines for the treatment of animals in behavioural research teaching (1997). The 3Rs are based on a humane approach to scientific experimentation and aim to: ‘Replace” animals used in experiments with non-sentient alternatives; “Reduce” the number of animals employed; and “Refine” animal experiments so that they cause minimum pain and distress.

**Figure 1:**
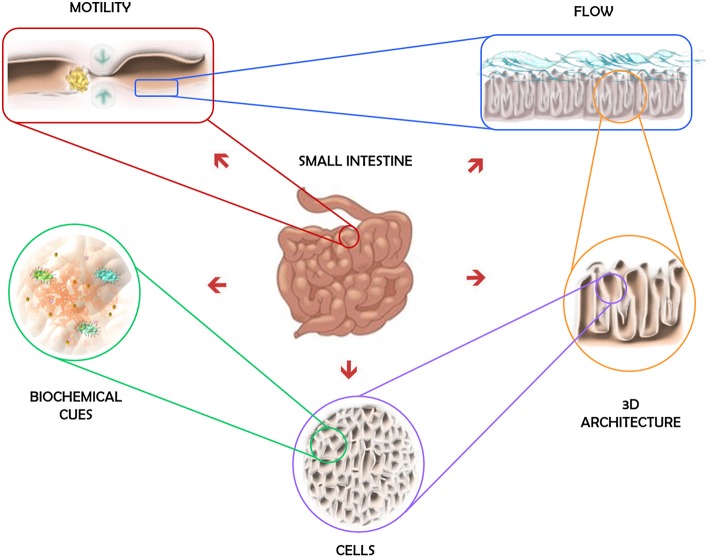
Key elements of the intestinal microenvironment.

The physiological environment of the intestine is characterized by its variegated composition, numerous functions and unique dynamic conditions. It is the organ responsible for the digestion and absorption of nutrients, but it also has secretory and immune functions (DeWitt and Kudsk, 1999; Santos and Perdue, 2000; Jaladanki and Wang, 2011). The intestinal epithelium, the most external layer of the mucosa, is the most self-renewing tissue of adult mammals and it includes different cell types, each of them specialized in a different function: enteroendocrine cells, Paneth cells, goblet cells, enterocytes, and microfold (M) cells (Crosnier et al., 2006). Roughly 90% of the absorption in the digestive tract happens in the small intestine (Balimane and Chong, 2005), since its epithelium folds into microscopic highly vascular finger-like projections, called villi. Besides providing an increase in the area available for the absorption of nutrients, the crypt-villus architecture is determinant in the migration of the intestinal epithelial cells as they differentiate (Heath, 1996). Moreover, the functions of the gut rely on the motility of its different parts. The motion itself has important functions, such has mixing, propulsion, and separation of luminal contents, that are the result of the coordinated interaction of excitatory and inhibitory neurons of the enteric nervous system (Chang and Leung, 2014). Additionally, the intestine is also where commensal microbes mainly live and interact with gut lymphoid tissues and the host immune system, which strongly influences intestinal homeostasis (Round and Mazmanian, 2009). The features of the intestinal epithelium's environment are schematically represented in Figure 1.

It is widely accepted that cell differentiation and proliferation are dictated by a combination of not only chemical, but also mechanical cues. Studies have demonstrated that mechanical factors, including mechanical forces, substrate stiffness, nanotopography of the adhesion surface and fluid flow can guide stem cell fate (Mousavi and Hamdy Doweidar, 2015). Likewise, the mechanical cues to which the intestinal epithelium is exposed should not be disregarded when developing a predictive intestinal model. Epithelial cells have exceptional interactions with their microenvironment, being subject to biomechanical and cues biochemical that determine cell fate and even contribute to pathological processes (Helmlinger et al., 1997; Farge, 2003; Keller et al., 2003; Brancaccio et al., 2006). This phenomenon is defined as mechanotransduction, i.e., how cells sense physical forces and then translate them into biological responses (Paluch et al., 2015). In recent decades much effort has been dedicated to understanding the mechanisms regulating mechanotransduction as manifested by the interest in providing mechanical stimuli to *in vitro* cell cultures.

One of the challenges of science fields relying on experimental cell biology and tissue engineering is the development of methods and strategies for implementing physiologically relevant *in vitro* models in order to better mimic tissue or organ responses. Before designing experiments, scientists must select appropriate culture conditions, define cell numbers and assess the tools available for creating appropriate *in vitro* models capable of answering the research questions posed. Some key parameters for the design of biomimetic *in vitro* intestinal models will be discussed in this review. To this end, we will start by defining the design specifications for an intestinal *in vitro* system. What does constitute an ideal intestinal model from an engineer's point of view?

An ideal *in vitro* model recapitulates all the essential features of the biological counterpart it is intended to represent. Considering the microenvironment of the intestine and examining the dynamic conditions to which epithelial intestinal cells are exposed, the ideal engineered human intestinal *in vitro* model (for healthy tissue) should contain the following features (summarized in Table 1):

- human-derived cells from all types representative of the native gut epithelium (ideally, also from all the layers of the mucosa) with the ability of being cultured for the defined assay time without losing their characteristics;
- a substratum with a 3D structure [villi-like and crypt structures, where the villi present a surface density of 10–40 mm^2^ and have a height between 0.5 and 1 mm (Hasan and Ferguson, 1981; Standring, 2016)] and similar properties to the native lamina propria in terms of its chemical composition and biomechanical behavior [elastic modulus around 0.5–1 kPa (Stidham et al., 2011; Stewart et al., 2018)];
- a fluidic system that provides adequate oxygenation and nutrients to the cell medium, as well as physiological shear stress [around 0.0002–0.008 Pa (Kim et al., 2012)];
- a flexible substrate to provide cycle deformation to the epithelium [around 8–10% strains at 0.15 Hz (Kim et al., 2012; Cei et al., 2016)];
- a biochemical environment that comprises the epithelium/immune system crosstalk (Araujo et al., 2016) and, of great importance, the microbiota of the gut [the approaches developed to address this last aspect are reviewed elsewhere (Wang et al., 2018)];

**Table 1:** Summary of some physiologically relevant parameters characteristic of the intestinal epithelium microenvironment.

**Stiffness**	**Shear stress**	**Strain**	**Villi**
0.5–1 kPa	0.0002–0.008 Pa	8–10% at 0.15Hz	Density: 10–40 mm^2^;
			Height: 0.5–1 mm

Is it possible to recreate all these features *in vitro*?

In an attempt to answer this question, this review is aimed at presenting the current methodologies used to develop intestinal *in vitro* models: from the most simplistic and traditional cell monolayer to the complex dynamic 3D models developed with the aid of engineered systems. The organization and discussion of the following contents reflect our critical view of the different solutions and their applications from a bio-engineering perspective.

## Cells

The intestinal epithelium is a tissue of particular interest as it is in constant cell renewal from the stem cells of the crypts. The crypts, together with the fibroblasts of the mucosa, form a niche of pluripotent stem cells that generate the main cell types—of which enterocytes are the most predominant. The phenomena which determine phenotype commitment and cell-specific expressions are not fully understood, thus the field attracts intense biological investigations (Simon-Assmann et al., 2007; Noah et al., 2011; Clevers and Batlle, 2013).

Inevitably, the cellular composition of any *in vitro* model is the main determinant of its outcomes. Choosing the cells to represent the human intestine outside of its physiological environment dictates the classification and applications of the *in vitro* model, as described in this section.

### The “What's Good Lasts Forever” Choice: Immortalized Cell Lines

Caco-2 cells are the most widely used cell model to study the permeability of drugs over the last 20 years. There are other cell lines that were reported to constitute intestinal *in vitro* models [reviewed in (Simon-Assmann et al., 2007)], but the Caco-2 line has been accepted as standard for prediction of drug intestinal permeability in humans by pharmaceutical companies and regulatory authorities (Hidalgo et al., 1989; Hilgers et al., 1990). They are an immortal human cell line derived from a human colorectal adenocarcinoma that is regularly used in the models of the intestinal epithelium. When in culture, they grow into a confluent monolayer and then differentiate, adopting a behavior very similar to enterocytes (Smetanová et al., 2011).

Caco-2 cell differentiation starts when the cells achieve confluence, around 7 days after seeding and is completed within 21 days. That is when the cells are polarized and connected to each other through tight junctions, exhibiting an apical brush border structure with the expression of several enzymes, transporters and receptors (Antunes et al., 2013a).

Nevertheless, the Caco-2 monoculture does not contemplate other important factors that influence the functionality of enterocytes such as the mucus layer or the interactions between the epithelium and the stroma (Li et al., 2013), pushing scientists to develop more complex cellular models. Furthermore, in different perspective, the most widely accepted cell culture protocol requires about 3 weeks, which can be labor intensive and time consuming, limiting its wide application in high-throughput screening of new compounds (Cai et al., 2014). Addressing this, some groups have developed modified Caco-2 culture techniques that require a shorter culturing period (Chong et al., 1997; Lentz et al., 2000; Sevin et al., 2013) by changing experimental conditions such as the medium composition and seeding density (Cai et al., 2014). Interestingly, the presence of flow consistently accelerates Caco-2 differentiation and barrier formation (Kim et al., 2012; Giusti et al., 2014; Cacopardo et al., 2019).

As a further improvement, a cell model consisting of a co-culture of Caco-2 and mucus producing HT29 cell lines was developed. It mimics both enterocytes and goblet cells, and it was reported to generate more predictable experimental results, due to the role of mucus on drug transport (Pontier et al., 2001; Mahler et al., 2009; Pereira et al., 2015b). Other models were developed to include the presence of M-cell-like cells that resemble the intestinal M cell—characterized by few irregular microvilli and elevated transcytotic activity (Clark et al., 2001; Araujo et al., 2016). Some researchers have also reported that Raji B cells can promote M cell phenotype in some Caco-2 cells (Kerneis, 1997; Gullberg et al., 2000; Araújo and Sarmento, 2013). Based on the rationale behind these two previous models, Sarmento's group developed a triple co-culture model (Caco-2, HT29-MTX and Raji B) that was able to induce the M cells phenotype in some cells and, additionally, to provide a higher transport of insulin (Antunes et al., 2013b; Araújo and Sarmento, 2013).

### The “Live Fast and Die Young” Choice: Primary Cells

As already mentioned, ideally, an *in vitro* model that closely resembles the human intestinal epithelium should comprise a combination of the different gastro intestinal (GI) epithelial cells able to be cultured for long periods.

Several attempts have been made to isolate and expand viable intestinal epithelium in *in vitro* cultures from human tissues. For instance, Lahar et al. (2011) developed a protocol that required various growth factors as well as the intimate interaction between the intestinal epithelial cells intestinal and sub-epithelial myofibroblasts (ISEMFs) to sustain epithelial growth *in vitro* and *in vivo*. Aldhous et al. obtained intestinal epithelial cells from duodenal biopsies together with Lamina propria (LP) cells. The epithelial cells were cultured on collagen membranes on top of LP cells and allogeneic Epstein-Barr Virus (EBV)-transformed B lymphocytes, in a 12-well tissue culture set-up (Aldhous et al., 2001).

In spite of all the efforts, a reproducible protocol for maintaining long term primary cell cultures from intestinal tissue samples remains a challenge (Lukovac and Roeselers, 2015), despite the fact that organoid technology has somewhat modified the research landscape.

### The “I Want It All” Choice: Intestinal Organoids or Mini-Guts

**Figure 2:**
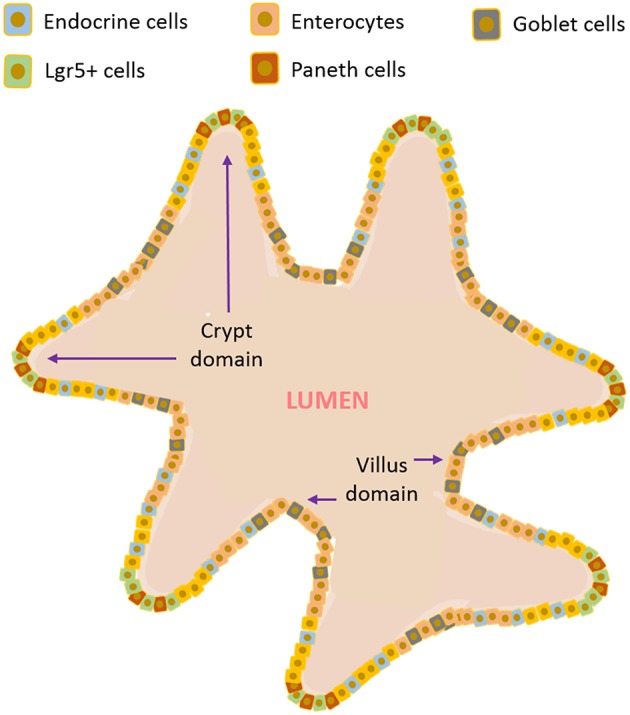
Illustrative scheme of a gut organoid.

In 2007, the identification of intestinal stem cells - the Lgr5 stem cells of the small intestinal and colonic crypts by Clevers and co-workers came as a “game changer” (Barker et al., 2007). Since these cells can differentiate into all intestinal epithelial cells (including also stem and progenitor cells), they can be grown *in vitro*, for longer periods, forming the so called “mini guts,” or organoids (Sato et al., 2009, 2011). These intestinal organoids are self-organized in 3D structures that recapitulate major features of native tissue, exhibiting a highly folded epithelium structure consisting of crypts and villi, just like the native intestinal epithelium. The organoid, illustrated in Figure 2, is composed of a central hollow region and several protruding structures. The external parts resemble the crypt structure of the small intestine, with Lgr5+ stem cells and Paneth cells in the apex region, while the central sphere consists mainly of differentiated cells (Nakamura and Sato, 2018). The obvious potential of these “self-sufficient” organoids in the field of regenerative medicine was instantly clear and the “mini guts” were successfully tested for engraftment in mice (Shaker and Rubin, 2012; Liu et al., 2016). The use of organoids is a fast growing field and it has been explored for other applications ranging from regenerative medicine (Wiegerinck et al., 2014) to host-microbe interaction studies (Lukovac et al., 2014) and from drug testing to disease modeling (Baumann, 2017). Nevertheless, organoids also have their shortcomings. Specifically, by themselves they are unable to mimic the biomechanical forces that stem cells are exposed to *in vivo*. Furthermore, since they are heterogeneous in terms of shape, size and viability and their 3D spatial arrangement limits drug penetration, they are usually unsuitable for drug screens (Fatehullah et al., 2016; Yin et al., 2016) and permeability studies. Remarkably, to overcome this issue some groups have developed enteroid monolayers, which are epithelial cells derived from the same Lgr5 stem cells but organized in 2D. These monolayers provide a structure for the basolateral addition of other cell types, such as immune cells or pathogens, and to study specific responses to different apical stimuli (Ettayebi et al., 2016; Braverman and Yilmaz, 2018). In such systems of course, the importance of a three-dimensional structure to better mimic the native tissue is neglected in favor of the desired application of the model—this is an aspect that will be discussed further ahead.

## 3D Architecture

**Figure 3:**
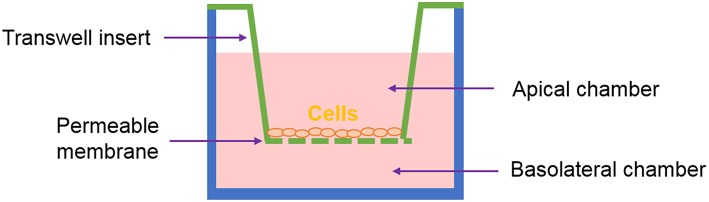
Representation of the Transwell® system.

One of the most common systems used to recreate *in vitro* the intestinal interface is the Transwell®, represented in Figure 3. It has been used to study intestinal permeability of drugs, toxins or microorganisms. Here, the epithelial cells are seeded on a membrane housed on a “transwell insert.” The insert separates the apical compartment, corresponding to the intestinal lumen, from the basal one - which represents the blood vessels. This system mimics the barrier configuration of the *in vivo* intestine to a certain extent (Pereira et al., 2015b).

**Figure 4:**
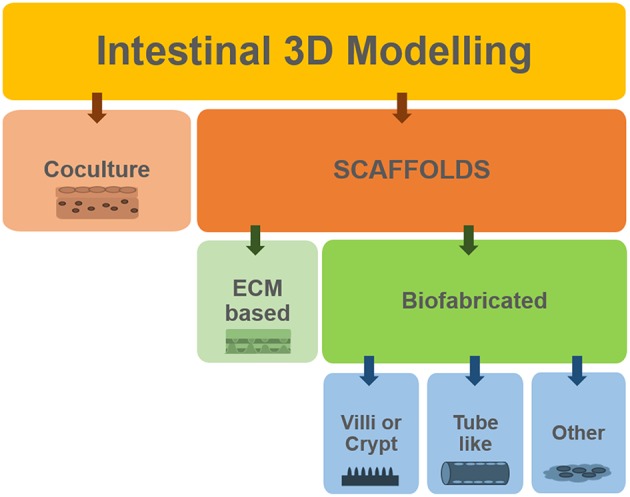
Summary of the different approaches used to develop 3D models of the intestine.

The models based on the Transwell® system benefit from high standardization and ease of use but they might be considered too reductionist. Certainly, most of the current understanding of many biological processes is based on studies conducted on two-dimensional (2D) monolayer-monotypic cultures, however cells *in vivo* exist in a 3D heterogeneous and complex environment which regulates cell activity. The intricate array of biochemical signals is controlled by the different cells as they produce and absorb molecules to/from each other and to/from the extracellular environment. 2D models lack the representation of these interactions and are a “reductionist approach” that does not properly represent the *in vivo* scenario. Therefore, 3D models are now considered as a means to bridge the gap between cells cultures and animal models (Pampaloni et al., 2007; Mattei et al., 2014). Figure 4 summarizes how we classify the methods employed for obtaining 3D models of the intestine, which will be described in the following sections. One can develop co-cultures organized in multilayers, where cells are deposited in different sheets, often embedded in an ECM-like substrate (such as Matrigel® or hydrogels). Alternatively, one can choose to use scaffolds. In general, the scaffolds obtained using biofabrication techniques are designed to mimic (or induce) either the villi/crypt architecture of the epithelium or the tube-like structure of the gut.

### Leveling Up: Multilayer Models

In recognition of the importance of matrix dimensionality, there has been a gradual shift from 2D monolayer cell cultures to 3D models. The multilayer models are a first step in this direction. Addressing the importance of stromal cells and extracellular matrix (ECM) in the homeostasis of intestinal epithelial cells (Powell et al., 2005), a couple of models were recently proposed comprising Caco-2 and goblet-induced HT29-MTX cells and stromal cells (Li et al., 2013; Pereira et al., 2015a) that better recreate the composition of the tissue. Another example is a co-culture system developed by Leonard and colleagues to study the inflammation process. The model consisted of a three-dimensional co-culture of human intestinal epithelial cells and immunocompetent macrophages and dendritic cells, which better recreate an increased inflammatory cytokine response (Leonard et al., 2010). Note that despite having a 3D rearrangement, and being thus more physiologically relevant, these models still lack the characteristic crypt-villi architecture.

### Cell Scaffolds: Sustaining the Idea of 3D

Besides organoids and multilayer models, the 3D structure is commonly achieved using a scaffold. Scaffolds are engineered structures that “aid” cells in organizing themselves similarly to the *in vivo* microenvironment.

Scaffolds can be fabricated in different ways and can be made of different materials (Bitar and Raghavan, 2012). Biological scaffolds derived from extracellular matrix (ECM) have been developed as substrates for remodeling of different tissues, including the gut. Small intestinal submucosa (SIS) scaffolds were also employed for generating *in vitro* models of the small intestine, particularly for their potential to induce stem cell differentiation. In a study by Schweinlin et al. (2016), intestinal organoids derived from intestinal crypts from healthy human small intestine were seeded on a decellularized SIS scaffold, in co-culture with fibroblasts. The organoids differentiated into different intestinal cell types after 7 days (this *in vitro* model also entailed the presence of medium flow as will be described in section To See Is to Believe: Monitoring). Again, using acellularized human tissue scaffold to culture bone marrow stem cells, Patil and co-workers (Patil et al., 2013) obtained differentiated epithelial cells and even reported endothelial cells repopulating the blood vessels of the scaffold, which also maintained its architecture with the villi intact and structural proteins.

#### Villi-Like Structure

The recreation of the 3D architecture of the intestinal epithelium has been the goal of numerous research groups. Some of them have focused on the reconstruction of the villi architecture through biofabrication techniques. For instance, in 2011, Sung et al. developed a 3D villi model consisting of a collagen hydrogel scaffold. The authors obtained the scaffold by a combination of laser ablation and sacrificial molding technique, using calcium alginate, which allowed the maintenance of both the complexity and integrity of the hydrogel structure. Evident morphological similarities were observed between the collagen scaffolds covered with the Caco-2 cells and the human jejunal villi (Sung et al., 2011).

Later, to evaluate the integrity and the role of the 3D model in predicting drug permeability, the same group adapted the collagen scaffold to an insert design. Maintaining Caco-2 cells on the scaffolds for 21 days did not shorten the villi, and also promoted the formation of multiple layers caused by cell penetration in the matrix as the collagen degraded. This led to a higher permeability of the tested hydrophilic drug, when compared with the data from 2D cultures, approximating the values obtained for mammalian intestines. Moreover, they reported that cell differentiation on the 3D scaffold varied along the villus (Yu et al., 2012). The research activity of the group led more recently to a comparative study on the absorptive and metabolic properties of Caco-2 cells cultured in the collagen hydrogel scaffolds and in monolayers. Cell growth was higher in the 3D villi model and its barrier function was similar to the *in vivo* scenario; the activity of the metabolic enzymes was also improved in the 3D model (Yi et al., 2017). These collagen cell scaffolds were also tested in a microfluidic device, an aspect which will be described in the next section (Shim et al., 2017).

Sharing the goal of mimicking the villi architecture, Costello et al. developed a synthetic 3D scaffold able to support the co-culture of epithelial cell types with selected bacterial populations. The study was directed at exploring microbe-induced intestinal disorders with the aim of developing targeted probiotic therapies. Poly lactic-glycolic acid (PLGA) scaffolds, with villi shape, were produced and seeded with Caco-2 cells. They were used as a model to mimic the adhesion and invasion profiles of certain bacteria species, as well as the therapeutic potential of two probiotics. The authors found that the 3D environment affected the probiotic action differently: while *Lactobacillus* was more successful at dislocating pathogens, *Escherichia coli Nissle* was more effective at hindering their adhesion (Costello et al., 2014b).

The same group employed the PLGA scaffolds to study cell behavior and drug absorption of the Caco-2 and HT29-MTX co-culture model. They observed that the two epithelial cell types on the scaffolds were able to mimic the morphology and differentiation profile verified in native intestinal tissue, as identified by the expression of differentiation markers and by mucus secretion (Costello et al., 2014a).

#### Inducing Villi/Crypt Organization

Other studies exploit scaffold technology for intestinal *in vitro* models not necessarily to recreate the villi architecture, but also to give cues to the cells to adopt a more *in vivo*-like behavior. In this context, a recent investigation by Dosh et al. aimed at investigating the potential of three different hydrogel scaffolds to support the 3D culture of Caco-2 and HT29-MTX cells and evaluate their ability to stimulate villi formation. Here, the authors used alginate hydrogels and cells were cultured in different set-ups as well as under static or dynamic conditions for up to 21 days. Caco-2 cell viability was increased when laid on the synthetic hydrogel scaffolds but diminished when suspended within them. In contrast, HT29-MTX maintained similar viability in both conditions. Furthermore, cells cultured in and on alginate scaffolds formed multilayer spheroid structures, whereas the cells layered on synthetic hydrogel scaffolds formed villus-like structures (Dosh et al., 2017).

The studies by Wang et al. (2014, 2017) also explored the recreation of the intestinal microenvironment for the differentiation of cells. More recently, a micropatterned collagen scaffold with a crypt-villus architecture and an adequate chemical gradient promoted the formation of a stem/progenitor-cell zone and supported cell migration along the crypt-villus axis (Wang et al., 2017).

By means of a different methodology, Kim and Kim (2018) developed an innovative process to print a human intestinal villi model, using a cell-laden bioink. Interestingly, the structure of the tissue was appropriately recreated, and the cells laid by this process presented higher activity and expression of differentiation markers with respect to the cells in the control (seeded using the traditional method).

#### “Tube-Like” Scaffolds

With a different perspective and on a different scale, Chen et al. established a 3D porous silk protein scaffolding system comprising an engineered hollow lumen. The hollow channel of the 3D scaffolds was used to house Caco-2 and HT29-MTX cells, while the porous bulk space was used to culture primary human intestinal myofibroblasts (H-InMyoFibs) embedded in a collagen gel. This scaffold and respective cell culture tubular architecture were shown to induce typical physiological responses by favoring accumulation of mucous secretions on the epithelium, leading to low oxygen tension in the lumen, and enabling interaction with bacteria from the gut. Moreover, this 3D intestinal model allowed maintenance of tissue function and cell phenotype for months (Chen et al., 2015).

Continuing the work of Costello's group on the PLGA scaffolds previously mentioned in this section, Shaffiey et al. investigated the growth and differentiation of intestinal cells on a novel tubular configuration scaffold. This time, the researchers used intestinal stem cells and tested the cell responses both *in vitro* and *in vivo* (with implantation in animal models). They report that the cells differentiated into crypt-villi structures on the scaffold and its colonization was enhanced by coculture with myofibroblasts, macrophages and probiotic bacteria. Remarkably, the implanted scaffolds enhanced mucosal regeneration *in vivo* (Shaffiey et al., 2016).

## Stressing (and Stretching) the Importance of Mechanical Cues for Cells: Bioreactors

This section describes some examples of engineered systems for mechanical stimulation of cells, with particular reference to the intestine. Cell monitoring is also considered.

Cells respond to mechanical stimulation in different ways, depending on the type, magnitude, frequency and duration of the stress applied. For instance, tensile and compressive stresses are applied perpendicular to the surface of the cell or 3D construct; shear stress is applied parallel to the cell or 3D construct surface; while strains can be applied by stretching an elastic cell substrate. To understand the effect of certain physical forces and deformations on cell and tissue behavior, physiologically relevant mechanical stimuli are applied at the cell and tissue level using devices known as bioreactors. These devices are purposely designed to control the temporal, spatial and intensity profiles of the force parameter (Lopez et al., 2008; Mattei et al., 2014).

Some techniques to study mechanotransduction phenomena in cells in their physiological microenvironment include the application of compression, tension, shear stress and hydrostatic pressure. As a more recent approach, the response of individual cells to mechanical stimuli has been explored. This research line uses advanced devices that are able to apply nano or micro-scale forces to individual cells or their constituents, such in the case of atomic force microscopy and traction force microscopy (García et al., 2007). Nevertheless, these very small-scale techniques might not be suitable for studying or mimicking the dynamics of a complex tissue and as such are beyond the scope of this review.

### To See Is to Believe: Monitoring

Advanced cell culture systems should allow the user to not only perform cell culture, but also to track it. Non-destructive and continuous readouts giving information on cell vitality and function are highly desirable characteristics for a cell culture bioreactor. Most biologists are dependent on visual monitoring of cells, as this provides an immediate assessment of morphology and cell numbers. Indeed, several devices for this purpose are composed (totally or partially) of transparent components (Cacopardo et al., 2019). Furthermore, since the intestinal epithelium is a physiological barrier, is crucial to assess the integrity and tightness of the epithelial cell monolayer in *in vitro* cultures. One way to do that is by measuring the trans-epithelial electrical resistance (TEER), which is the electrical, ohmic resistance of the cell layer (Benson et al., 2013). The higher the value of TEER, the more compact and integral is the barrier, indicating well developed tight cell-cell junctions which inhibit paracellular current flow. The most popular TEER measuring system is the Epithelial VoltOhmMeter EVOM (World Precision Instruments) that comes with chopstick-shaped electrodes for measuring the TEER in transwells. Due to the advent of more sophisticated cell culture systems, over the years researchers have been developing alternative measuring approaches that are adapted to different kind of devices (in terms of scale and measuring method). Of particular note is the transepithelial electrical impedance (TEEI), which, like TEER, provides information on the low frequency resistance associated with paracellular current flow as well as the high frequency impedance of the cell barrier which is related to transcellular membrane-mediated capacitive currents. Interfacing or integrating a TEER or TEEI measurement systems with cell culture devices that include the presence of dynamic components (flow and motility) can be an extra challenge, especially in the case of micro-fluidic bioreactors due to their small volume and available space. Nevertheless, several research groups have successfully fabricated fluidic systems for intestinal *in vitro* models that allow electrical measurements in different modalities, as discussed in some the cases illustrated in section Flow.

### Flow

**Figure 5:**
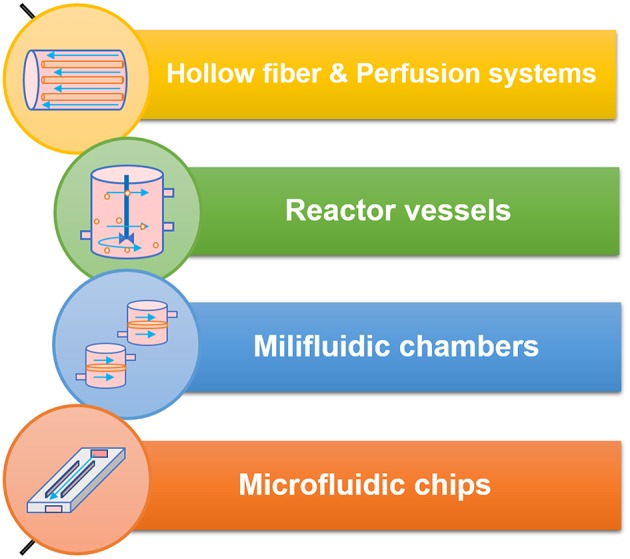
The different classes of bioreactors and flow systems and some of their technical specifications.

Controlled shear stress has been applied to cell cultures through different flow chambers, which use either peristaltic, pressure driven or syringe pumps that apply a parabolic laminar flow profile. When it comes to the systems developed to study the intestinal phenomena, we can divide them in five major classes as represented in Figure 5.

#### Hollow Fibers

Back in 1995, McBride et al. developed a bioreactor made of hollow fiber cassettes (McBride et al., 1995), that was later adapted in 1999 by the researchers to culture a cell line of human intestinal cells. The goal was to study the effect of chronic dietary or environmental toxin exposure. This set up allowed the researchers to perform a long-term study, in contrast with the acute toxic effect, back then usually verified in flask tissue culture (Hanley et al., 1999). Several years later, the hollow fiber bioreactor set-up is still applied to morphologically mimic the human small intestinal lumen. In 2013, Deng and his colleagues used porous hollow fibers of polyethersulfone (PES) to culture Caco-2 and study their differentiation and function. The study revealed an accelerated expression of Caco-2 cell function, suggesting its ability to simulate the original tissue microenvironment (Deng et al., 2013).

#### Go With the Flow: Perfusion Systems

Often, bioreactors are used to improve the culture conditions of primary cells, that are known for short survival times when cultured on the classic static systems. In order to sustain in culture intestinal organoids harvested from rats, Kim et al. developed a perfusion bioreactor. The results confirmed the survival of the cells seeded on the scaffolds and cultured in the bioreactor for 2 days (Kim et al., 2007). A work by Pusch et al., consisted in using decellularized porcine jejunal segments co-cultured with Caco-2 cells and primary human microvascular endothelial cells (hMECs) in a dynamic bioreactor. Some of the data were comparable with classic Caco-2 testing results, while some results demonstrated that in the decellularized segments cultured in the bioreactor cells resembled normal primary enterocytes and showed increased permeability to tested substances, when compared with static cultures (Pusch et al., 2011).

As mentioned in section Cell Scaffolds: Sustaining the Idea of 3D, Schweinlin and colleagues (Schweinlin et al., 2016) developed organoids using SIS that differentiated after 7 days. Additionally, the epithelial barrier function was tested under the effect of flow and with the co-culture of sub-epithelial fibroblasts as well. It was verified that the presence of the intestinal fibroblasts stabilized the barrier integrity and that the dynamic culture in a perfused bioreactor induced expression of differentiation markers on the epithelial cells, indicating that such complete system can sustain the culture of primary intestinal cells (Schweinlin et al., 2016).

#### The “Food Processor-Like”—Reactor Vessels

To better recreate the conditions of digestion, including even a food matrix, a dynamic GIT model was conceived in 2005 by Mainville et al (Mainville et al., 2005). It consisted of two reactors, one simulating stomach conditions and the other simulating the duodenal microenvironment, that would mimic the target organs, so that the authors could understand the interactions with the probiotics. The dynamic model was shown to better represent the events during upper GIT transit than conventional methods (Mainville et al., 2005). Six years later, Tompkins et al. adapted this system to perform similar studies on the same subject and were able to conclude that some probiotics should be ingested at a specific time interval from the meal (Tompkins et al., 2011).

Continuing with the theme of the research on probiotic bacteria, a gastrointestinal tract simulator (GITS) bioreactor was conceived by Sumeri et al. The system consisted of a fermentation vessel equipped with diverse sensors and different pumps to provide the flow of diverse fluids. The work established that such GITS could be successfully used for evaluation of viability of probiotics (Sumeri et al., 2008).

#### Millifluidic Chambers

Giusti et al. assessed a novel two-chamber millifluidic bioreactor for the culture of intestinal epithelial cells. After analyzing the fluidic dynamics and pressure gradients for different combinations of flow rates using computational models, Caco-2 cells were cultured on the device until they fully differentiated. The authors verified that the dynamic conditions led to an increase in barrier integrity values and in expression of tight-junction markers with respect to the static controls. Interestingly, the permeability of the cell barrier to the tested compound was higher in dynamic conditions, suggesting that the bioreactor could be used to perform drug delivery and nanomaterial toxicity studies on different barrier tissues (Giusti et al., 2014). Recently, Cacopardo et al described the design and development of a bioreactor with an integrated sensing system (embedded biocompatible electrodes interfaced with an impedance meter) that allowed for online TEER and TEEI measurements and the results obtained supported the finding of Giusti et al. (Cacopardo et al., 2019).

#### Microfluidic Devices

Microfluidic devices often designated “Organ-on-a-chips” are a trend nowadays. They were originally developed using technology adapted from semiconductor manufacturing and generally hold chambers perfused with culture media and colonized by cells. The cells are arranged to simulate tissue- and organ-level physiology (Bhatia and Ingber, 2014).

In 2008 Kimura et al. developed a micro pumping system on-chip. Caco-2 cells were cultured in the device for more than 2 weeks. Perfusion and transport measurements (using fluorescent compounds detected with an optical fiber system) were conducted, targeting the micro bioreactor for applications in toxicity testing and drug screening (Kimura et al., 2008).

In 2010, microfluidics was an established technology, as shown by the works of Imura et al. (2009, 2010). The authors developed a microchip-based system that mimicked the intestine. The microdevice was mainly composed of polydimethylsiloxane (PDMS) sheets, with microchannels fabricated using photolithography techniques, and the flow was regulated with a microsyringe pump. The results of absorption tests run on cultured Caco-2 cells in the microchip were consistent with those obtained using conventional methods, suggesting the suitability of the new system (Imura et al., 2009). In the following year, the same research group used the developed microchip to integrate micromodels of tissues—a component for the intestine (using Caco-2 cells) and another one for the liver (using HepG2 cells). The authors tested the intestinal absorption, hepatic metabolism, and bioactivity of different substances and claimed the feasibility of the operations on their device, reducing time and cell consumption compared to the classic *in vitro* assays (Imura et al., 2010). Some years after, with a similar approach, Bricks et al. joined cell culture inserts and microfluidic biochips in a fluidic platform to study the interaction between intestine and liver, using Caco-2 and HepG2, respectively. This work revealed that the integrity, viability and metabolism of both cell types were maintained and that the co-culture system allowed for biotransformation of a tested compound (Bricks et al., 2014).

Addressing the need to monitor the integrity of the intestinal epithelial barrier, some authors considered the measurement of TEER in their microsystems. Shah et al fabricated a microdevice, called HuMix, that served to study the host–microbe molecular interactions in the gut. One of its versions was designed to allow the insertion of a commercial chopstick style electrode to measure the TEER (Shah et al., 2016). With the purpose of studying the permeability of substances across an intestinal barrier, Tan et al fabricated a microfluidic device with two sets of electrodes embedded in the chip. To perform the TEER measurements in this device, the wires coupled to the electrodes were connected to a multimeter (Tan et al., 2018).

Microfluidics systems can also be used to recreate more than one organ, evolving into what we call body-on-chip designs, as it is the case of the next example. A modular GI tract-liver system by co-culture of primary human intestinal epithelial cells and 3D liver micro-lobe like construct was conceived by Chen et al for preclinical studies applications (Chen et al., 2018). In these systems the intestinal cells differentiated into major cell types of the native tissue and formed a monolayer displaying TEER values comparable with physiological values. Additionally, the permeability of the cell barrier obtained was compared to a conventional permeability model using Caco-2 cell response for drug absorption by measuring the uptake of standard substances.

#### Going Further With the Flow, Adding Architecture

Bringing together the presence of dynamic conditions and the fabrication of structures to mimic the intestinal architecture (in this case, crypts or villi), some authors have developed highly sophisticated systems to recreate the microenvironment of the epithelium.

Last year, Costello et al. developed *in vitro* artificial small intestines. Essentially, a small intestinal bioreactor was constructed using polymeric scaffolds that mimicked the 3D architecture of the tissue. The data obtained from the TEER measurements (since the device was interfaced with an EVOM) indicated that the presence of flow induces characteristics of cell barrier tightness which are closer to the physiological condition with respect to static culture. An increase in cell proliferation was also verified and the authors reported that some cell responses varied according to different regions of the construct and according to different tested flow rates (Costello et al., 2017).

Shim et al. (2017), integrated a collagen scaffold that mimics the human intestinal villi [developed originally in (Sung et al., 2011)] in a microfluidic device, where the absorptive permeability of the epithelium (composed of Caco-2 cells) as well as the activity of representative enzymes were determined. As hypothesized, the results suggested that the combination of fluidic stimulus and 3D structure can induce further improvement in the physiological relevance of intestinal *in vitro* models.

### Motility

As any other mechanical force, stretch can be an important modulator of cell physiology. For more than 40 years researchers have been developing systems to study cell/tissue stretching effects (Leung et al., 1977) that rely on the application of static or cyclic deformation to monolayers of cells cultured on deformable membranes or 3D scaffolds.

**Figure 6:**
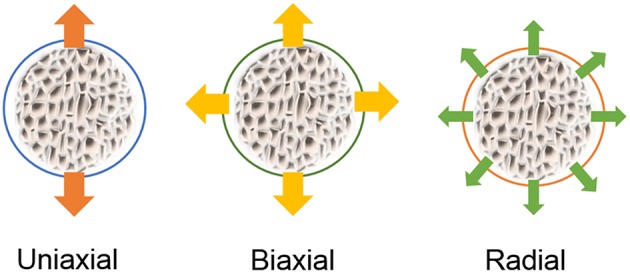
Representation of typical strain fields applied to cells on flexible membranes stretched in-plane.

These systems can vary in configuration, scale operation and mechanism of actuation, and there are many categories into which they can be grouped. One can divide them, for instance, in in-plane stretch systems and out-of-plane stretch systems, or even by the type of strains they provide: uniaxial, biaxial, or equiaxial (often referred as radial, when stretching circular shaped substrates) (Brown, 2000). Some versatile systems can also provide compression and not only stretching, as in the example of the *in vitro* model of the intestine using electroactive polymers as an actuating cell culture membrane (Cei et al., 2016). The most common types of strain applied in-plane are as illustrated in Figure 6.

Indeed, different studies have shown that cyclic mechanical stretch induces proliferation, increases tissue organization, and enhances mechanical properties on several cell types, as it briefly illustrated in the following section.

#### Engineering Peristalsis

When it comes to the intestine, the epithelium should be affected by the repetitive deformation during peristaltic distension and contraction and by the repetitive shortening of villi. In a notable work by Basson et al. Caco-2 cells were cultured on an elastic membrane and subjected to 10% strains (on average), applied with vacuum induced out-of-plane deformation, and it was verified that the cyclic strain stimulated proliferation. This response was higher in the membrane periphery where strain was maximal and, furthermore, it modulated the expression of specific brush border enzymes. The authors concluded that mechanically-induced strains at a physiological frequency and magnitude enhanced proliferation and modulated the differentiation of this cell line in an amplitude-dependent way (Basson et al., 1996). Later, the molecular pathways that led to the reported effects were investigated and the proteins PKC and tyrosine kinase were pointed as regulators of intestinal epithelium proliferation and brush-border enzyme activity upon cyclic deformation (Han et al., 1998).

Using a rat *in vivo* model, Safford and his colleagues suggested that mechanical tension induced intestinal growth. Their results indicated that the applied mechanical tension led to an increase in Paneth cells numbers, that caused proliferation and reorganization of the mucosa and *muscularis propria*. In addition, the increased intestinal length corresponded to an increase of enzymatic activity, suggesting a potential augmented absorption of the stretched bowels (Safford et al., 2005).

This time using an *in silico* model, more specifically a computational intestinal organoid culture model, Buske et al. studied the role of biomechanics on the stem cell niche formation in the gut. The researchers improved a previous computational model of intestinal tissue (Buske et al., 2011) by adding a flexible membrane that assigned a bending modulus to the organoid surface. According to the test results, the proliferation induced shape changes leading to the formation of crypt-like domains. The spontaneous localized tissue curvature could be contribute to the regulation of stem cell organization (Buske et al., 2012), illustrating once more the effects of cell deformation on tissue differentiation.

Back to *in vitro* models of Caco-2 cells, a more recent study by Samak et al. explored the impact of cyclic stretch on tight junction and adherens junction integrity. The results indicated that due to activation of specific signaling pathways, those apical cell junctions were disrupted, as supported by the re-organization of the junction's proteins and increased paracellular permeability (Samak et al., 2014).

Currently, perhaps one of the most advanced *in vitro* models of the human intestine is the “human gut-on-a-chip” by Kim et al. It consists of a microfluidic device that contemplates both the shear stress induced by fluid flow and the cell stretching induced by a deformable membrane. The device is composed of two microfluidic channels separated by a porous flexible membrane coated with ECM that was seeded with Caco-2 cells. The microenvironment of the intestinal epithelium was mimicked by using a medium flow at a rate of 30 μL h^−1^ that produced a shear stress of 0.02 dyne cm^−2^ and by applying cyclic uniaxial strain (10%; 0.15 Hz) that imitated physiological peristaltic motions. This system allowed for a quick polarization of the epithelium that spontaneously grew into folds and formed a high integrity barrier (Kim et al., 2012).

Later, this same system was used to co-culture commensal microbes in contact with the intestinal epithelium cells. After 1 week, the authors report that immune cells and endoxins together stimulated epithelial cells to produce proinflammatory cytokines which can induce villus injury and compromise intestinal barrier function. This showed that the chip can also be used to study interaction between microbiome and intestinal pathophysiology in a controlled environment (Kim et al., 2016).

More recently, Kasendra et al. (2018) developed a small intestine-on-a-chip for culturing epithelial cells obtained from intestinal biopsies that were previously expanded as 3D organoids and then dissociated and culture on the porous membrane of the chip in co-culture with human intestinal microvascular endothelium. This device was conceived to provide both flow and uniaxial cyclic deformation to the cells. The authors reported the formation of villus-like projections lined by polarized epithelial cells which differentiated similarly to the organoids in terms of cell linages, but this time exposing their apical surfaces to the lumen-like channels. Moreover, transcriptomic analysis indicated that the chip closely mimicked the human duodenum *in vivo* with respect to the precursor organoids.

## Discussion

In this paper we identified some of the key physical parameters that characterize the microenvironment of the intestinal epithelium and that are therefore desirable in an *in vitro* model. A number of technologies and systems which recapitulate these features are described and summarized in Table 2. As can be seen from Table 2, many of them address a couple, or even several, of the requirements for the “ideal intestinal *in vitro* model” (listed in the Introduction and distilled in Table 1).

**Table 2:** Summary of the current and emerging tools for intestinal *in vitro* models and their typical applications.

**MODEL TYPE**			**3D**	**Motility**	**Flow**	**Interface**	**Microflora**	**Examples**	**Applications**
Classic culture systems	*Cell line based*	*Classical models*	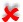	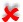	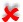	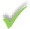	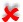	Hilgers et al., 1990	Permeability and toxicity studies
		*Advanced models*	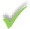	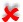	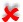	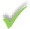	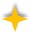	Mahler et al., 2009; Pereira et al., 2015a	Differentiation studies; Permeability and toxicity studies
	*Native tissue like*	*Short term explants*	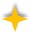	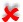	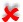	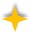	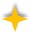	Aldhous et al., 2001; Lahar et al., 2011	Differentiation studies; Cell interaction; Tissue engineering;
		*Organoids*	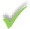	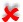	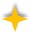	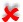	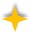	Sato et al., 2011; Yin et al., 2016; Baumann, 2017	Differentiation studies; Pathogenesis; Tissue regeneration;
Engineered culture systems	*Microscale*	*3D structures*	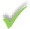	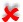	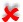	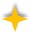	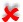	Wang et al., 2017; Kim and Kim, 2018	Differentiation studies
		*Microfluidics/Gut-on-a-chip*	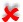	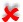	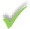	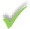	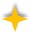	Imura et al., 2009; Bricks et al., 2014; Shim et al., 2017	Differentiation studies; Permeability and toxicity studies; Host-microbe interaction
		*Deformable membrane*	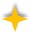	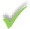	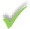	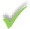	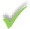	Kim et al., 2012; Kasendra et al., 2018	Differentiation studies; Host-microbe interaction
	*Macroscale*	*Scaffolds based*	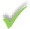	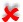	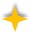	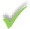	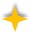	Yu et al., 2012; Costello et al., 2014b; Dosh et al., 2017	Differentiation; Permeability studies; Tissue regeneration
		*Flow systems*	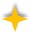	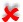	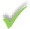	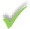	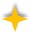	Mainville et al., 2005; Pusch et al., 2011; Deng et al., 2013	Differentiation studies; Permeability studies
		*Deformable membrane*	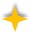	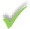	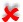	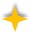	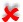	Basson et al., 1996; Samak et al., 2014; Cei et al., 2016	Differentiation studies

*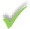, Typically yes; 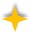, In some cases yes; 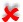, Typically NO*.

As the complexity of the biomimetic models increased, they better approximated the *in vivo* tissue. For instance, as mentioned in section Stressing (and Stretching) the Importance of Mechanical Cues for Cells: Bioreactors, the model of gut-on-a-chip by Kim et al. (2012) integrated the presence of fluid flow, cyclic peristalsis-like movements, commensal microbes and the presence of villi-like 3D structures (whose formation was induced by the microenvironment). This device is indeed one of the finest examples of the success of the organ-on-chip technologies. However, since the chip only comprises one layer of the intestinal wall, it is not enough to study phenomena such as inflammatory diseases, that are modeled by the interaction with nervous and immune systems cells (Vasina et al., 2006). In contrast, the work by Kasendra et al. (2018) used a similar approach but pursued a higher complexity at the cellular level, but then again, it did not include the presence of the microbiota. One interesting aspect of this work was the use of “disassembled organoids,” showing that despite being a breakthrough in this field, organoids do possess some limitations. Although one can have the representation of all the cell types of the epithelium, the interactions with non-parenchymal cells is still lacking. Furthermore, as seen previously in section Cells, due to their architecture, the cells cannot be exposed to mechanical stimuli and are not easily available for drug transport studies (Fatehullah et al., 2016; Yin et al., 2016). Nowadays, the microfluidics technology explored in these two last examples is of great popularity, but it still has a long road of improvement ahead, such as the standardization protocols and eventually, the adaptation/integration into human-on-a-chip systems for pharmacokinetic and pharmacodynamic studies. Conversely, one might ask: is the microscale enough to recreate the complexity of a tissue? Some authors prefer using miniaturized bioreactors that deal with milli-fluidics, arguing that microfluidic devices contain few functional units—groups of cells which can mimic organ function at the micro-scale–thus, they are not representative of a tissue. Additionally, microscaled devices present “extremely high surface to volume ratio, which gives rise to high wall shear stresses,” and prompt the “so-called edge-effect, with a large portion of cells lying at the periphery of the system and not interacting properly with the bulk of the cells” (Mattei et al., 2014).

Indeed, the recreation of the microenvironment of the cells is of major importance, as was underlined in section 3D Architecture. It is expected that emergent biofabrication techniques will allow for the recreation of the complex environment cues of the intestinal mucosa, by creating heterogenous constructs with different biochemical gradients for different cells (Malda et al., 2017). Another goal in the tissue engineering field is the promotion of vascularization and intestinal models are no exception. With an adequate blood vessels network, the *in vivo* tissue can be better represented, and appropriate supply of nutrients is ensured for the 3D cell structures thicker than 100–200 μm (Du et al., 2011).

So far, it seemed that the merging of all the presented features in one single system was the best approach to obtain more predictive outcomes from an intestinal *in vitro* model. However, the question arises: is it really necessary? Or even better: when is it necessary? The answers may vary, but it appears to be consensual that the ideal features of a model depend on its usage.

Certainly, the applications for tissue engineering require different conditions compared to those required for other fields—as was verified in the studies that started from a 3D cell organoid and opted for a monolayer configuration n of the epithelium. The 3D architecture of a tissue is definitely desirable for regenerative applications, ideally counting on the presence of constituents of other layers of the mucosa as well. But it might not be ideal if the scope of the study is to assess the permeability of a recently discovered compound, for instance.

As a concluding remark, the design criteria of an intestinal *in vitro* model depend on its application. Having identified the application, a researcher should refer to the relevant physiological parameters, such as those listed in Table 1 (when applicable) to develop an appropriate model. Table 2 may be useful to identify the most suitable tools that can be used to develop such models focusing on their features and typical applications.

## Author Contributions

All authors listed have made a substantial, direct and intellectual contribution to the work, and approved it for publication.

### Conflict of Interest Statement

The authors declare that the research was conducted in the absence of any commercial or financial relationships that could be construed as a potential conflict of interest. The handling editor declared a shared affiliation at the time of review, though no other collaboration with the authors.
